# The Employers' Liability Act and the Hospitals

**Published:** 1903-04-11

**Authors:** 


### Editor's Letter-Box.

[Our Correspondents are reminded that prolixity is a great bar to publication, and that brevity of style and conciseness of statement greatly facilitate early insertion.]

### THE EMPLOYERS' LIABILITY ACT AND THE HOSPITALS.

*To the Editor of* The Hospital.

P. Michelli, Esq., Seamen's Hospital, Greenwich, S.E., writes: The Employers' Liability Act of 1897 has had the effect of diverting contributions from voluntary hospitals. At first sight there would appear to be reasonable ground for employers of labour to refuse to contribute to these institutions, as they insure against injury to their workpeople and no longer feel the need of the hospitals. The Employers' Liability Insurance Societies, in like manner, argue that as they have to pay compensation for injured employees the latter must find their own medical aid. But this is a superficial view of the subject and overlooks the fact that the hospital enables the insurance companies to charge a lower rate of premium and frequently reduces the amount they have to pay in compensation, while the employer pays a lower rate of premium than would be the case if there were no hospitals. These facts are apparent when it is remembered that it is owing to the prompt treatment afforded in the hospitals that a lacerated hand is saved from amputation and the insurance company pays less for a lacerated hand than an amputated one. In like manner a life lost costs the insurance company a large amount, but, if saved, is a gain to the company and a reduction of premium to the employer. The existence of the Employers' Liability Act should stimulate both the companies and the employers to support the hospitals, but unfortunately the opposite has been the result. It is not fair that both insurer and insured should profit at the expense of the hospitals, and I would suggest that each company be asked to pay a percentage on profits or premium to these charities. As there may be some difficulty in apportioning the amount, it might be paid to King Edward's Hospital Fund.

